# Computer Modeling of the Structure and Spectra of Fluorescent Proteins

**Published:** 2009-07

**Authors:** A.V. Nemukhin, B.L. Grigorenko, A.P. Savitsky

**Affiliations:** 1 Department of Chemistry, M.V. Lomonosov Moscow State University;; 2 N.M. Emanuel Institute of Biochemical Physics, Russian Academy of Sciences;; 3 A.N. Bach Institute of Biochemisty, Russian Academy of Sciences

## Abstract

Fluorescent proteins from the family of green fluorescent proteins are intensively used as biomarkers in living systems. The chromophore group based on the hydroxybenzylidene-imidazoline molecule, which is formed in nature from three amino-acid residues inside the protein globule and well shielded from external media, is responsible for light absorption and fluorescence. Along with the intense experimental studies of the properties of fluorescent proteins and their chromophores by biochemical, X-ray, and spectroscopic tools, in recent years, computer modeling has been used to characterize their properties and spectra. We present in this review the most interesting results of the molecular modeling of the structural parameters and optical and vibrational spectra of the chromophorecontaining domains of fluorescent proteins by methods of quantum chemistry, molecular dynamics, and combined quantum-mechanical-molecular-mechanical approaches. The main emphasis is on the correlation of theoretical and experimental data and on the predictive power of modeling, which may be useful for creating new, efficient biomarkers.

## INTRODUCTION

**Fig. 1.**
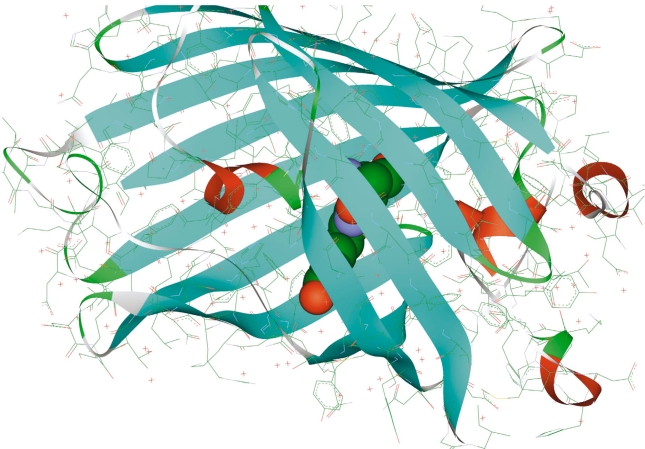
Structure of GFP (PDB ID: 1EMA). The chromophore group is emphasized

**Fig. 2.**
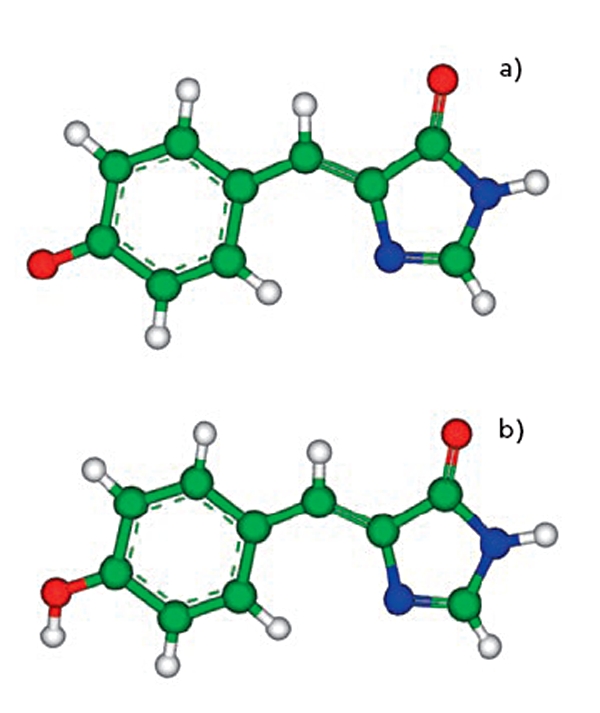
The chromophore molecule of GFP. Top (a): the anionic form; bottom (b): the neutral form. Here and below, carbon atoms are distinguished by green, oxygen atoms by red, and nitrogen atoms by blue

The discovery and use of colored proteins from the family of the green fluorescent protein [1-7] stimulated an avalanchelike growth of interest in these amazing species. Their practical value is explained by their ability to label cell clones with colored proteins and then literally trace the inner cell events. Biotechnology perspectives are promising because of multicolor labeling, in particular, the ability to observe interprotein interactions in living systems. These proteins are well characterized in crystallography studies. The β-sheets form the walls of the can (Fig.1) which efficiently shield the chromophore from the external media. The latter is represented by the hydroxybenzylidene-imidazoline molecule (Fig.2), which is formed in nature from three amino-acid residues inside the protein globule. The photophysical properties of fluorescent proteins are explained by transformations occurring with this chromophore group inside the macromolecule upon light illumination at certain wavelengths.

Researchers from different fields concentrate on studies of all the aspects of the structure and mechanism of fluorescent proteins. In this review, we primarily analyze works on the computer modeling of the structure and spectra of these species. Using modern tools of molecular modeling [8] may provide considerable support to experimental studies, allowing one to save time and resources for a comprehensive examination of the processes occurring in such complex molecular systems. Obviously, a description of the transitions between the electronic states of chromophore molecules responsible for light absorption and emission requires the use of the quantum theory, correspondingly, quantum chemistry is an appropriate modeling tool. The conformational states of the protein macromolecule and the structure of the chromophore- containing domain are also important for the properties of fluorescent proteins, thus requesting the application of molecular mechanics and molecular dynamics methods. To employ all these approaches, substantial computer resources, as well as efficient numerical algorithms and computer programs, are necessary.

Quantum chemistry models are based on a nuclear-electron picture of a molecular system that requires a numerical solution of the Schroedinger equation by using approximations of different accuracy levels. Presently, a developed hierarchy of quantum chemistry approaches is known, each of which is oriented toward performing certain tasks. In particular, for calculating structural parameters, i.e., geometrical configurations corresponding to the minimum energy points on the ground electronic state potential energy surface for a given model molecular system, as well as to calculate vibrational spectra, electronic density functional theory approaches are often operative. Multiconfigurational wavefunction approximations are preferable (e.g., [9]) if excited state parameters are requested, which includes calculations of transition energies for estimates of band positions in the optical spectra or the location of the conical intersection points. Software packages of quantum chemistry, including GAUSSIAN, GAMESS, MOLPRO, NWCHEM, TURBOMOLE, are used in practical applications.

The potential energy surfaces which are supposed to be directly calculated in quantum chemistry models are approximated by analytical functions in the methods of molecular mechanics and molecular dynamics. These analytical functions include chemical bond stretches, valence angles and torsional angles deformations, interactions of chemically unbound atoms, electrostatic contributions, and, sometimes, other terms. Each contribution of such kind is represented by an expression with parameters (the so-called force fields), the adjustment of which is the goal of numerous research groups. The most popular force field parameters suitable for modeling protein systems are included in the AMBER , CHARMM, OPLSAA, and GROMOS libraries, as well as others.

A certain breakthrough in the molecular modeling properties of biomolecular systems is accounted for by the development of the combined methods of quantum- and molecular mechanics (QM/MM). According to the main idea of this approach [10], the smaller fraction of the protein macromolecule, in which electronic redistributions or transitions between electronic states are assumed to be important, is included into the quantum subsystem. The energies and forces in the latter are computed by using different approaches of the quantum theory. The vast majority of the protein atoms surrounding such a selected central part are assigned to the molecular mechanical subsystem described by the force field parameters. In QM/MM approaches, the energy of each point on the potential surface is computed as a sum of the energy of the quantum part immersed in the field of the MM subsystem and the molecular-mechanical energy itself. An analysis of such composed potential energy surfaces allows us to investigate the photophysical properties of the chromophore inside the protein.

Below, we consider the most interesting results of a molecular modeling of structural parameters, the optical and vibrational spectra of the chromophore containing domains of fluorescent proteins by methods of quantum chemistry, and molecular dynamics and combined quantum- and molecularmechanical approaches. The main emphasis is on the correlation of theoretical and experimental data and on the predictive power of modeling, which may be useful for the creation of new efficient biomarkers.

## Modeling the Structure and Dynamics of Fluorescent Proteins Using Classical Force Fields

The macromolecules of the fluorescent proteins contain aminoacids for which the force field parameters are well presented in most conventional libraries of molecular mechanics and molecular dynamics (MD). However, for the chromophore itself formed upon the cyclization of aminoacides in the presence of molecular oxygen, nonconventional types of atoms occur.

**Fig. 3.**
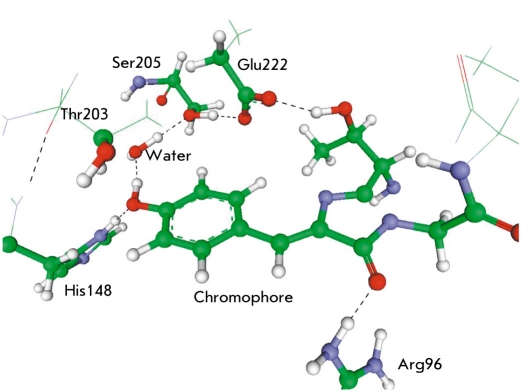
Hydrogen bond network (black dashed) near the GFP chromophore. Labeling amino acid residues corresponds to the structure PDB ID: 1EMA

Reuter et al. [11] reported the parameters compatible with the CHARMM force fields for the molecule 4′;-hydroxybenzylidene- 2,3-dimethylimidazolinone representing the GFP chromophore that were adjusted by the results of quantum chemical calculation. The first works [11, 12] on molecular dynamical simulations with relatively short classical MD trajectories for the wild type and mutated variants of GFP were carried out using the heavy atom coordinates of crystal structures 1EMA and 1EMB from the Protein Data Bank [13]. It is worth commenting that the atomic coordinates that are deposited in this database following the results of X-ray or NMR experimental studies often are preliminarily refined by computer calculations by molecular-mechanics-based software. Simulations allow one to add missing hydrogen atoms in the model structures of protein macromolecules, although the known uncertainties appear, first and foremost, for the histidine, glutamate, and aspartate. In [11, 12], the rigidity of the protein globule was demonstrated. Also, the hydrogen bond networks near the chromophore both in the neutral and the anionic forms were reported. To emphasize the value of these data, we show in Fig. 3 the hydrogen bond network near the GFP chromophore obtained in our own calculations.

The known optical properties of GFP [14] exhibit two main absorption bands at 400 nm and 480 nm for the wild type of the protein. These bands are associated with the chromophore either in the neutral state (form B in Fig. 2b) with a shorter wavelength or in the ionized form (form A in Fig. 2a) with a longer wavelength. Since the hydrogen-bond network should provide routes for proton transfers connecting these two forms (presumably through the intermediate form I), its modeling has attracted much attention since the very first works. In this section, we mention only those theoretical papers which describe the applications of classical models.

In particular, the role of rotation of the Thr203 side chain (Fig. 3), which presumably facilitates the transition between the forms A and B, has been analyzed with the molecular dynamics simulations [15]. The papers [16, 17] describe possible proton transfer routes between various forms of the chromophore, taking into consideration water molecules and the nearest amino-acid side chains following detailed molecular dynamics simulations. Papers [18, 19] discuss the consequences of quite extended proton migration over the hydrogen bond networks (up to the exit to the protein surface) for an interpretation of the photophysical properties of GFP.

**Fig. 4.**
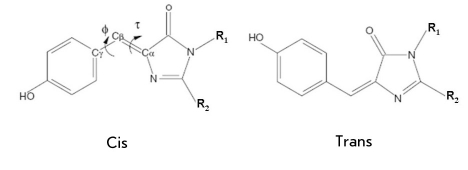
Cis-trans isomerization of the GFP chromophore

Another important transformation in fluorescent proteins, namely, the cis-trans conversion of the chromophore (Fig. 4), was analyzed by molecular mechanics [20-22] and molecular dynamics [23-25] methods. Such cis-trans isomerization may be of great value for the so-called blinking colored proteins, in which fluorescent states appearing for finite time intervals alternate with dark states, depending on external factors. The principal working hypothesis to explain the mechanism of such behavior is based on the suggestion of the cis-trans chromophore isomerization inside the protein until the fluorescent state is reached and quenched. In the forthcoming sections of our paper devoted to the results of quantum-based calculations, this hypothesis is also discussed.

One very interesting result in modeling the cis-trans chromophore isomerization inside the protein environment that also illustrates the modern achievements of classical molecular dynamics simulations was reported in a recent paper [25]. The authors computed free energy profiles as the profiles of mean force for the GFP chromophore along the internal rotation angle (Fig. 4) at a temperature of 300 K inside the protein matrix of the Ser65Thr mutant. All protein atoms and almost 9,000 solvent water molecules were included into the model system. By using the results of quantum chemical calculations, the authors modified the parameters of the AMBER force field in such a manner that they might be assigned to the excited electronic state. The biased MD simulations were applied to drive the φ rotation in order to remove the system from the regime of small oscillations around the minimum energy point and to scan the extended regions of the configuration space. The calculation shows that ~8 kcal/mol is required to overcome the energy barrier and provoke the cis-trans chromophore isomerization along the coordinate φ.

Paper [23] describes the results of classical MD simulations for the trans-cis isomerization of the chromophore in another colored protein asCP (or asFP595) [26], for which kindling fluorescence is observed. This phenomenon means that the initially nonfluorescent protein form may be transformed into a form with red emission by intense green light illumination. Presumably, the photoinduced trans-cis chromophore isomerization is responsible for such behavior. Trajectory calculations [23, 27] that have been performed with OPLSAA force field parameters allow one to visualize the possible movements of the chromophore and the nearest amino-acid residues upon speculating processes.

Methods of classical MD were used in [28] to study the possible cis-trans chromophore isomerization in the ground electronic state of the protein Dronpa [29], for which the lightinduced switch from the fluorescent state to the dark state was observed. As in the case of other photoswitchable color proteins, the hypothesis that the chromophore isomerization was responsible for the adjustment of the photophysical properties of the protein was verified. The authors of [28] used the AMBER force field modified by new parameters for the chromophore molecule by the results of quantum chemical calculations. It was shown that the chromophore group resided in the cis conformation; however, point mutations on the positions of the nearest amino-acid residues might enhance the flexibility of the protein macromolecule.

Not long ago, a research group from the bioengineering and bioinformatics department of Moscow State University used classical MD simulations to model the structural features of the monomeric red fluorescent protein mRFP1 upon point mutations on position Glu66.

At the end of this section, we note the important role of molecular mechanics and molecular dynamics methods in modeling the conformational states of proteins. Estimates of equilibrium atomic coordinates and an analysis of the time evolution of geometric parameters in protein macromolecules containing several thousand atoms can be practically performed only within classical mechanics by using empirical or semiempirical force fields. Since the conventional forcefield parameters from the AMBER and CHARMM libraries are well calibrated to describe hydrogen bonds, an apparent achievement of such modeling for the fluorescent proteins is the picture of the hydrogen bond network in the chromophore- containing domain. On the contrary, the computed energy parameters, such as the internal rotation energy barriers upon cis-trans chromophore isomerization in the ground state and, especially, in the excited state, should be considered with caution, taking into account the high sensitivity of the results to the ambiguously defined force-field parameters for these properties. The qualitative conclusions that can be drawn from the results of MD simulations, i.e., the time evolution of the hydrogen bond network, crude estimates of energy barriers upon conformational changes accompanied by the movements of peptide groups, or the chromophore provide valuable information. However, for more accurate estimates, which may include proton transfers over hydrogen bond networks, as well as for an analysis of potential energy surfaces in the ground and excited states, the quantum calculations should be addressed.

## Quantum Chemistry of Chromophores in the Gas Phase and in Solutions

**Fig. 5.**
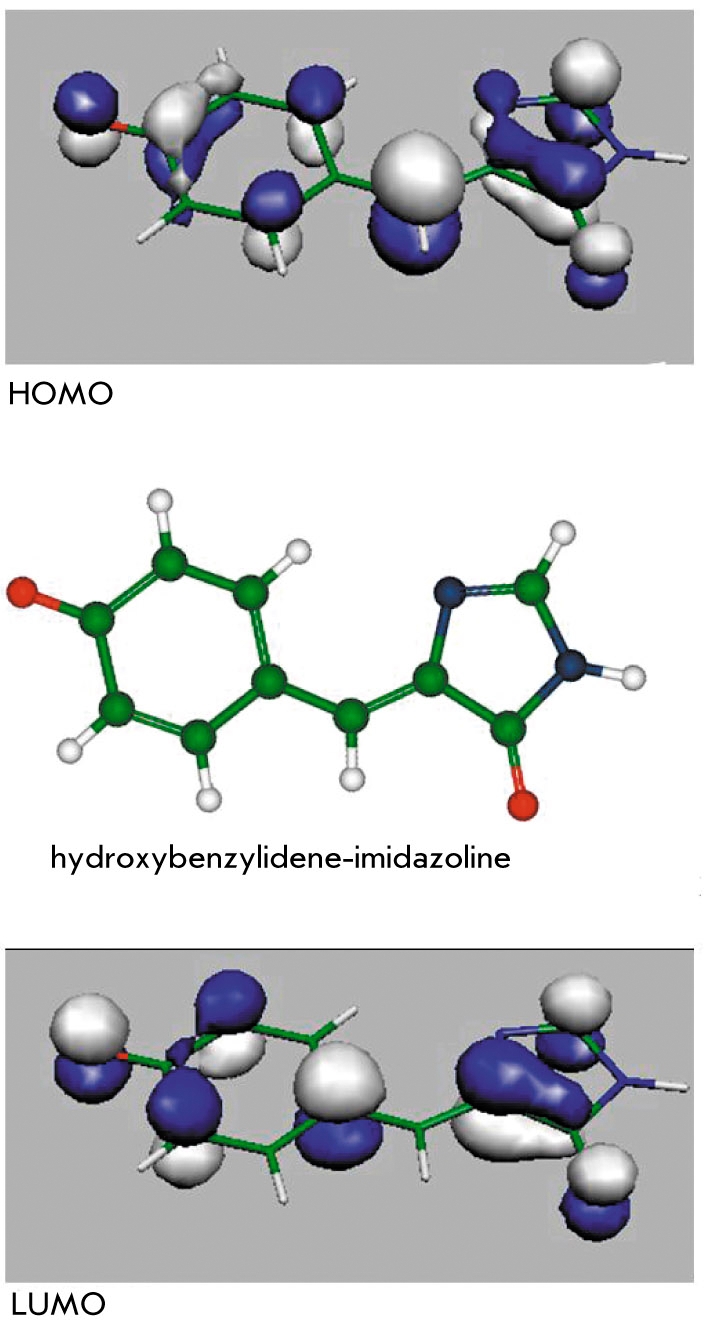
Middle: the hydroxybenzylidene-imidazoline molecule in the cisanionic form representing the GFP chromophore; bottom: the highest occupied molecular orbital (HOMO); top: the lowest unoccupied molecular orbital (LUMO)

The very first quantum chemical calculations of the electronic structure of the model chromophore molecule of GFP [31-34] allowed one to assign the light-induced electronic excitation (photoabsorption) to the transition between singlet states S0 → S1. In terms of orbitals, this transition corresponds to the electron transfer from the highest occupied molecular orbital (HOMO) of the π-type to the lowest unoccupied molecular orbital (LUMO) of the π*-type. Figure 5 illustrates images of these orbitals for the anionic form of the molecule 4′-hydroxybenzylidene-imidazolinone (see also Fig. 4) calculated in [35]. According to these results, the electronic excitation affects the local properties of the electronic density in the bridging region connecting the phenyl and imidazolinone rings of the chromophore molecule. As a consequence, the parameters of the initially single (C-CH) and double (-CH=C) chemical bonds (in the ground state) become more alike, thus facilitating internal rotation over the angle τ (Fig. 4) around the initially double bond.

The choice of a strategy to provide accurate calculations of the most important quantitative properties of the chromophore groups of the colored proteins from the GFP family is constantly under discussion among specialists in computational quantum chemistry. These properties include the energy differences upon excitation (S0 → S1) and descent (S1 → S0), which are associated with the band maxima in the excitation and fluorescent spectra and the corresponding band intensities, as well as the sections of the ground and excited state potential energy surfaces needed for interpreting the phototransformations of the chromophore groups. The first review of earlier calculations is most likely due to Helms [36]; one of the most recent discussions of the achievements of quantum chemistry for the chromophores in vacuo is presented [37]. The methodology aspects of quantum chemical approximations suitable for modeling photochemical processes with organic molecules are clearly presented in the review articles [9, 38, 39]. To avoid getting mired into quantum chemistry terminology and the details of different approximations used presently for computer calculations of the properties of organic chromophores in the ground and excited states, we limit ourselves to a superficial description of the most common approaches. Several commonly used abbreviations will be used below.

Currently, it is accurate to state that the calculations of equilibrium geometry parameters in the ground electronic state for molecules composed of up to a hundred atoms are not problematic. By using the methods of the density functional theory (DFT), a large community of chemists can calculate the three-dimensional structure of the chromophore molecule and visualize its details on the screen of a monitor with suitable software.

**Fig. 6.**
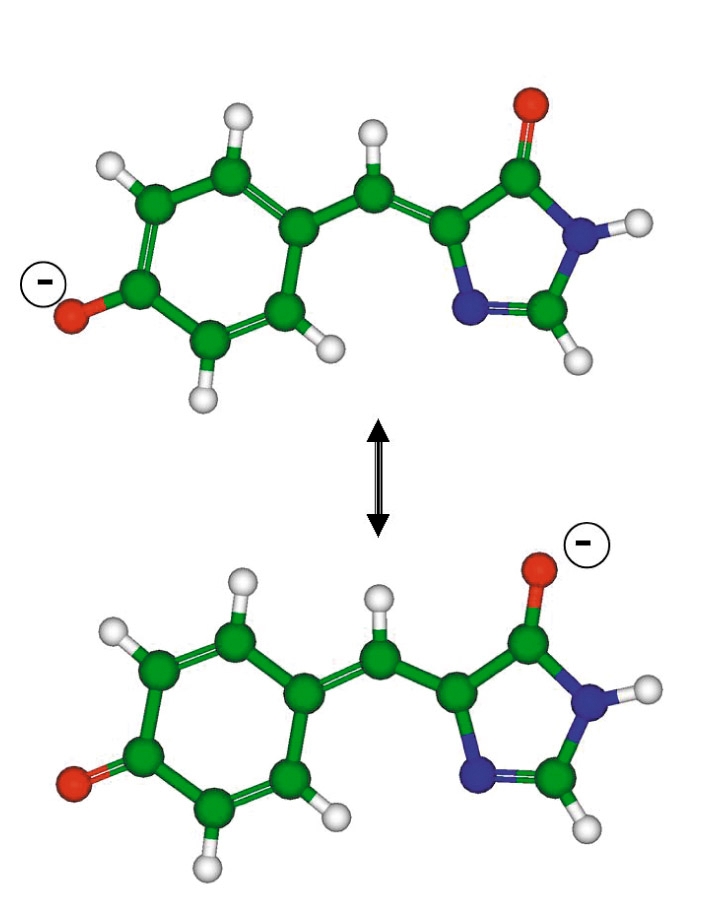
Resonance structures of the anionic form of the GFP chromophore

The difficulties of modeling optical spectra are due to the necessity of maintaining a similar accuracy level when calculating the ground state electronic properties with a leading electronic configuration …π2 and those of the excited state with a leading electronic configuration …π1π*1 (three dots ahead of the π-type HOMO refer to the entire set of preceding orbitals doubly occupied by electrons). It would be beneficial to take into account the superposition of electronic configurations for the ground electronic states as well. The reasons for such a description are clear, e.g., when considering the resonance structures for the anionic form of the GFP chromophore (Fig. 6). Correspondingly, the quantum chemical approaches with the multiconfigurational wavefunctions seem suitable for calculations. The complete active space self-consistent field (CASSCF) method is one approach often met in papers devoted to the photochemistry of organic molecules.

The calculations with multiconfigurational approaches require practical skills, access to powerful computational resources, and patience in waiting for the results; therefore, the temptation arises to resort to shortcuts. In the first papers devoted to the calculation of the absorption spectra of the chromophores from the fluorescent proteins [31-33, 40, 41], as well as in recent papers [27, 42, 43], fairly good results for the band maxima in the optical spectra and band intensities are often obtained with simple semiempirical methods like ZINDO [44]. In this method, considerable simplifications of the electronic structure theory are balanced by the successful adjustment of parameters using suitable reference experimental data. As always happens with semiempirical methods, it is unclear a priori when their application may be successful and when they will lead to large errors.

The current state of things regarding another modern calculation method of the energy differences between the ground and excited electronic states which is becoming more and more popular among quantum chemists due to its simplicity - the time-dependent density functional theory (TD-DFT) - is even more complicated. In many applications, TD-DFT leads to excellent agreement with the experimental data for the positions of band maxima in the optical spectra of organic chromophores, but in other applications the results are considerably less ambitious (see, e.g., [35]). There are fundamental reasons for this failure [45] that are due to errors in describing the charge-transfer states that are common for such molecules.

We can compare the achievements of these two "user friendly" calculation methods of band parameters in the absorption spectra of the chromophores of colored proteins, ZINDO, and TD-DFT. According to the first paper [41] devoted to studies of the red fluorescent protein DsRed [46] (in particular to modeling properties of its chromophore), the results of ZINDO are closer to experimental findings than those of TD-DFT. In a recent paper [42], the authors compared the computed spectral parameters of the anionic forms of the chromophores from GFP and DsRed with those measured by using photodestruction spectroscopy in the gas phase [47-49]. The experimental value for the absorption band of the anionic GFP chromophore is 479 nm, while calculations with ZINDO result in 477-481 nm and the position of the intense absorption band calculated with TD-DFT (390 or 405 nm, depending on computational details) deviates considerably from the experimental value. For the anionic form of the model chromophore synthesized by the motives of the DsRed chromophore, the position of the experimental absorption band is 521 nm [49], and calculation results are 533 nm (ZINDO) and 449 nm (TD-DFT). A similar conclusion follows in [43], which is devoted to studies of the anionic GFP chromophore: the ZINDO method allows one to obtain a position of the absorption band that practically coincides with the experimental value, while the TD-DFT method overestimates the vertical excitation energy, giving rise to a blue shift from the experimental estimate (479 nm) by 50-90 nm. Nevertheless, we stress again that the predictions of the semiempirical ZINDO method should be taken with caution. It is unclear how to systematically improve ZINDO, unlike the TD-DFT approach, for which sooner or later more reliable representations of the electronic density functional will be found. Meanwhile, new publications appear that report the results of excitation energy calculations for the chromophore molecules from different-colored proteins in different versions of the TD-DFT approach [27, 35, 37, 50-55].

Let us turn to methods on the grounds of multiconfigurational approaches, which are more creditable in the quantum theory - but less "user-unfriendly" - and the use of which requires substantial computer resources and experience in quantum chemical calculations. Potentially, these methods are necessary for solving a wider range of problems than calculations of the absorption bands of the chromophores. Namely, the multiconfigurational approaches are used to compute sections of the potential energy surfaces of the excited electronic states with the proper localization of the minimum energy points needed to estimate fluorescence spectra. Also, they are used to locate the points of conical intersections of the ground and excited states where quenching of photoexcitation occurs.

Using the less developed [34] and more sophisticated [37, 55, 56] versions of the so-called configuration interaction methods - in which superposition of electronic configurations takes place for calculations of energy differences between the ground and excited states of the chromophore molecules in the gas phase - allows one to achieve, in favorable cases, estimates for the optical band positions with errors not exceeding 20-50 nm. Additional efforts (see, for instance, [9, 38, 39] for details) are spent on optimizing the orbitals entering the multiconfigurational wavefunctions to make these orbitals suitable "on average" for the ground and excited electronic states and for the optimal choice of the number of orbitals occupied by electrons in the ground and excited state. Thus, we arrive to the CASSCF method with state-averaging, SA-CASSCF, which seems to be the most basic one for calculating the excited state potential surfaces of organic chromophores. For better accuracy, the SA-CASSCF energies are corrected by adding the perturbation energy contributions. After such corrections, the errors upon estimating the band maxima in the optical spectra of the gas phase chromophores are reduced to 15-20 nm. Examples of these state-of-the-art calculations are presented in [37, 57, 58] for the GFP chromophore and in [59] for the asFP595 chromophore. Papers [58, 59] include results for different protonation states of the chromophore molecule.

**Fig. 7.**
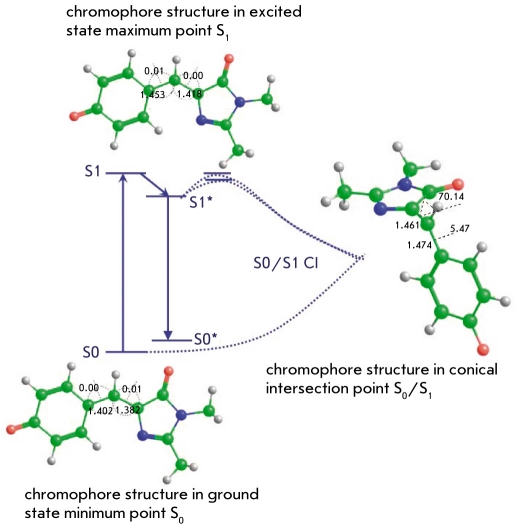
Possible transformations of the GFP chromophore in the cis-anionic form upon photoexcitation

Figure 7 illustrates possible transformations of the chromophore molecule upon photoexcitation, taking the GFP case as an example. Upon transition from the ground state S0 minimum energy point to the potential energy surface of the excited state S1, the system relaxes to the energy minimum responsible for fluorescence. The drift on the excited state potential energy surface can lead to the conical intersection point S1/S0 with a distinct geometry configuration through which descent to the ground state occurs.

This picture, which provides important information on the photophysical processes with chromophore molecules, can be reliably obtained with quantum chemistry methods on the basis of the SA-CASSCF approach. The first such study for the anionic form of the GFPchromophore in vacuo was reported in [57]. Later, the calculations for the gas phase anionic chromophores in both cis- and trans-conformations from the green (GFP) and red (DsRed, asFP595) proteins were described [60-62], which helped rationalize the chromophore photoisomerization processes.

Beyond calculations of excited states, quantum chemistry methods have been used for computations of structural parameters, vibrational spectra, and for analyzing possible re-arrangements in the ground electronic state in model systems composed of the chromophore with the nearest molecular groups by motives of the protein structure. The first such calculations for a fairly large molecular cluster mimicking the chromophore containing domain of GFP are described in [63]. By using the cluster model, the authors of [64] also calculated the proton transfer pathways along the hydrogen bond network (see Fig. 3) for the chromophore containing the domain of GFP in the ground electronic state, concluding that the activation barriers for these transitions should not be large. The vibrational spectra of the GFP chromophore in various protonation states were computed in [65] by using the Car-Parrinello molecular dynamics, which presents a quite popular methodology based on estimates of the forces acting on a nuclei by solving quantum equations in the density functional theory. In [66], the authors considered a larger model for the chromophore-containing domain of GFP. A direct comparison was performed for the Raman spectra calculated for the chromophore molecule and those measured experimentally for GFP. By using the same methodology, the bands in the Raman spectra of the DsRed chromophore were computed in [67]. The results of calculations of the vibrational spectra of the GFP chromophore molecule in different protonation states are reported in [68]. Despite a certain practical use for the calculation results of the vibrational spectra of a small model system composed of the gas-phase chromophore molecule and several of the nearest peptide groups, the disadvantages of such an approach are also evident. These disadvantages are related to the insufficient inclusion of the protein environment. In this respect, the results of approaches in which solvent effects are taken into account, as in [69, 70], seem to be more interesting.

Modeling chromophore molecules in solutions by quantum chemistry tools presents an important step in studying the effects of the condensed phase environment on chromophore properties. These studies are usually performed either within the continuum model, treating the solvent as a media with a specific value of the dielectric constant in whose cavity a solute species is inserted, or within the discrete model with an explicit consideration of the solvent molecules in the model system. 

The continuum solvation model is used in paper [71], along with the semiempirical calculation method of excited state energies [31] for estimates of the solvent-induced shifts in ethanol in the optical spectra of the GFP chromophore in different protonation states. A qualitative correlation between the theoretical and experimental data was obtained.

Important results were obtained in paper [72], in which the diagram illustrating the photo-induced transformations with the neutral form of the GFP chromophore (see Fig. 7) was calculated for a model system composed of a chromophore surrounded by water molecules. In this work, the sections of potential energy surfaces for the ground and excited states were computed and the coordinates of minimum energy points and conical intersection points were located. The semiempirical quantum chemistry method AM1 with parameters specially adjusted for this project was used to perform such complex calculations. The main conclusion of this paper, which is widely cited in the literature devoted to studies of fluorescent proteins, is that the solvent reduces the lifetime of the excited electronic state of the chromophore over the gas phase by an order of magnitude. Unlike in the gas phase conditions, the internal rotation of the chromophore over the bridging double bond (Fig. 4) is facilitated inside the shell of solvent molecules. In [73], the molecular dynamics of the neutral form of the GFP chromophore surrounded by water molecules was studied using the ab initio quantum chemistry approach SA-CASSCF for potential surfaces. It was concluded that the solutions have increased quenching efficiency compared to the gas phase process.

Papers [70, 71] describe calculations of the vibration spectra and of energy profiles for the quenching of the photoexcitation of various protonation forms of the GFP chromophore in aqueous solution. The continuum solvation models, i.e., the polarized continuum model (PCM) was used, and the ab initio computation quantum chemistry methods on the basis of CASSCF for the potential surfaces were applied. The increased efficiency of internal conversion in solvent was also confirmed.

Another approach to the model properties of the modified GFP chromophore in the cis- and trans-conformations in various protonation forms in an aqueous solution was demonstrated in [52]. The distribution of particles in the model system composed of the chromophore and the solvent shell of 857 water molecules were simulated by the Monte Carlo method for the NPT ensemble. The excited state energies of the chromophore were computed in the TD-DFT and CASSCF approaches, the latter being recognized as the better choice. The solvent shifts in the absorption spectra and the cis-trans isomerization options of the chromophore in solution were analyzed. Similar methodology was later used for studies of the DsRed chromophore [74].

The authors of [55] calculated the absorption spectral band maxima for a series of the GFP-type chromophores with changes inside the chromophore molecule itself by using the continuum PCM model and different versions for estimates of the excitation energies. It was concluded that there is fairly poor agreement between theoretical and experimental data, although qualitative correlations could be established. Synthetic molecules on the basis of the GFP chromophore were also studied experimentally in aqueous solution in [75], accompanied by theoretical estimates for the optical spectra by the TD-DFT method and contributions from the solvent within the PCM approach.

The optical spectra of the chromophore 2-acetyl-4-(phydroxybenzylidene)-1-methyl-5-imidazolone from the protein asFP595 were studied experimentally in several solvents at different pH values. In aqueous solution, the band at 418 nm was assigned to the neutral form, and the band at 520 nm was assigned to the anionic form of the chromophore. Band positions in ethanol, propanol, and dimethylformamide were found to be considerably shifted with respect to water, and no correlation was observed with the corresponding values of the solvent dielectric constant. Simulations of these spectra were carried out for different protonation states of the chromophore in the cis- and trans-conformations in water, ethanol, acetonitrile, and dimethylsulfoxide (DMSO) [35]. The PCM continuum model and the TD-TDF approach for calculations of excitation energies were applied. The data collected in Table 1 illustrate the relationship between the experimental and theoretical results. The qualitative correlation is evident - both investigations establish a weak dependence band position on the solvent. The assignment of the shorter absorption band to the neutral form and the assignment of the longer absorption band to the anionic form are also apparent, although the quantitative disagreements are fairly large (up to 50 nm). The experimentally observed spectra cannot be definitely assigned either to the trans- or the cis-conformation of the chromophore in solution. The energy calculations for both conformations in vacuo and in the solution clearly predict that the energy of the cis-form is lower by about 1.5 kcal/mol than that of the trans-form. These calculations do not confirm the hypothesis formulated in [76] that the weak fluorescence of the chromophore observed in dimethylformamide is evidence of the similarity between the optical properties of this solvent and those of the protein asFP595.

**Table 1:** Comparison of calculated [35] and measured [76] (bold, in parentheses) wavelengths for the absorption band maxima of the chromophore asFP595. The asterisk distinguishes the wavelengths measured in DMF (ε=38.3)

Solvent	Neutral form	Anionic form	Zwitterionic form
Cis-conformation			
Vacuo (ε=1)	430	484	521
Ethanol (ε=24,3)	453 (425)	504 (542)	538
Acetonitrile (ε=36,3)	453 (422*)	502 (572*)	537
DMSO (ε=47,2)	458 (422*)	511 (572*)	545
Water (ε=80)	453 (418)	502 (520)	537
Trans-conformation			
Ethanol (ε=24,3)	438 (425)	476 (542)	504
Water (ε=80)	437 (418)	474 (520)	538

**Fig. 8.**
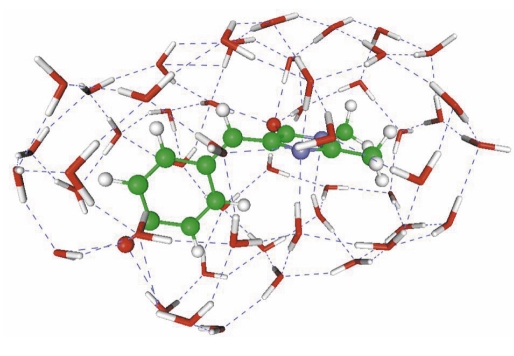
Structure of the transition state of the anionic GFP chromophore on the way from the cis-isomer to the trans-isomer inside the shell of water molecules

The question of whether or not the cis-trans isomerization of chromophores from the colored proteins in solutions is possible has been debated for a long time [77]. The experiments described in paper [78] show that the GFP chromophore, e.g., in the anionic form, can be transformed from one conformation to another with an activation barrier of about 13 kcal/mol. The latter was estimated by kinetic measurements using the Arrhenius equation. On the other hand, earlier quantum chemical calculations [33] resulted in barriers greater than 50 kcal/mol. This discrepancy was resolved only recently. In [79], the energy profile for the cis-trans isomerization of the anionic GFP chromophore in water was calculated to be 10-11 kcal/mol, correspondingly, which is very close to the experimental estimates. This theoretical result was obtained within new versions of the continuum solvation models and within the discrete model with an explicit treatment of water molecules in the first salvation shell. Figure 8 shows the structure of the model system in the conformation on the top of the activation barriers upon transition from the cis-isomer to the trans-isomer. Paper [79] underlines the necessity of using multiconfigurational approaches of quantum chemistry to adequately describe the isomerization energy profile.

Finally, we mention one more important application for the computer modeling properties of chromophores from fluorescent proteins in solutions, namely, calculations of pKa′s. This information is of value for an analysis of the chromophore properties in protein matrices since it helps estimate the chromophore′s protonation states and on-the-proton transfer pathways over hydrogen-bond networks. The pKa values are computed using the thermodynamics cycle components, which include the free energies of deprotonation on specific atoms in the gas phase and free energies of solvation of the protonated molecule, the anion, and free energy of proton solvation. A series of quantum chemical calculations should be carried out to obtain the parameters of the particles, including equilibrium geometry parameters and vibrational frequencies in the gas phase and in solution (in the latter case, with the continuum solvation models). Such a procedure and the corresponding results for the oxygen and nitrogen centers of the GFP chromophores are described in [80-82]. In [81], the pKa′s for the excited state were estimated as well. The computed pKa′s for the chromophores of colored proteins asFP595 and zFP538 in the trans- and cis-conformations are presented in [82].

To conclude this section, we stress the necessity of quantum chemical calculations for modeling the properties of the chromophores from the fluorescent proteins, despite their high cost. Upon improving the computational methods of quantum chemistry, the latter will become more and more user-friendly. The routine of calculating geometric structures (equilibrium geometry parameters in the ground electronic state) for molecules with a number of atoms up to 100 serves as an illustrative example - the user can obtain fairly reliable results on personal computers even without clear knowledge of the algorithms. So far, such a service is not available for modeling the entire process of photoexcitation; however, the situation may change with time.

## Modeling the Properties of the Fluorescence Proteins using the QM/MM Method

Modeling properties of the chromophores inside the protein matrix should be carried out using a combination of the quantum- mechanics and molecular-mechanics (QM/MM) methods. Apparently, the chromophore molecule itself should be assigned to the quantum subsystem by placing the boundary between the QM and MM parts in such a manner that all the conjugated bonds responsible for light absorption and emission are described by quantum equations. It should also be reasonable to include the side chains of amino-acids nearest to the chromophore molecule in the QM part, because they can be involved in the proton transfer process with the chromophore.

**Fig. 9.**
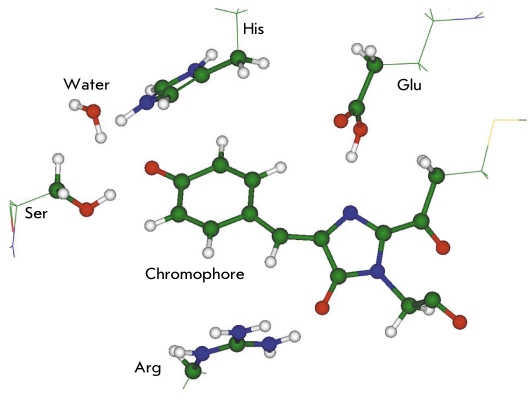
One possible choice of the quantum subsystem (balls and sticks) for modeling the properties of fluorescent proteins from the GFP family by using the QM/MM method

For practical purposes, the size of the quantum subsystem may amount to up to a hundred atoms. Figure 9 illustrates the possible choice of the QM subsystem for the QM/ MM calculations of the protein properties of the GFP family. The chromophore group (here it originates from the protein asFP595) is represented in the QM part almost as a whole. The side chains of Glu, His, and Ser, as well as the water molecule, may participate in proton transfers. The positively charged side chain of Arg may considerably affect the quantum subsystem.

With such a selected model, calculations of the structures and energies for the chromophore-containing domains from the asFP595 protein by considering both the trans- and cisconformations of the chromophore were performed in [83, 84]. The largest part of the protein macromolecule including more than 2,000 atoms surrounding the quantum subsystem was assigned to the MM subsystem. The initial coordinates of heavy atoms were taken from the crystal structure of the dark form of the protein PDBID:1XMZ [85] with the chromophore in the trans-conformation. After the hydrogen atoms (or protons) were added, the equilibrium geometry configuration of the model protein system was calculated with the flexible effective fragment QM/MM method [86, 87] using the Hartree-Fock approximation in the quantum subsystem and the AMBER force field for the molecular mechanic part. The obtained atomic coordinates were consistent with the crystal structure [83, 84]. Then, a model system was prepared in which the cis-conformation of the chromophore inside the protein matrix and the coordinates were re-optimized with the QM/MM method. One of the most important results of this study was the conclusion that the energy of the system with the trans anionic chromophore was lower than that with the cis-conformations. In vacuo, the ordering of conformations is reversed and the cis-isomer of the isolated chromophore should be lower than the trans-isomer. Therefore, the protein matrix provides greater stabilization for the transisomer of the chromophore, which is in agreement with Xray studies [23, 85, 88]. Estimates of the vertical excitation energies in the quantum subsystem using the TD-DFT method were performed for the structures optimized with the QM/ MM method. It was found that the structure with the cisconformation should correspond to the transition S0-S1, with a longer wavelength in the optical spectra. This result is also qualitatively consistent with experimental observations and with the working hypothesis [23, 26, 89, 90] according to which asFP595 absorbs green light in the state with transconformation of the chromophore and emits red light in the state with cis-conformation of the chromophore.

Attempts to theoretically describe the mechanism of kindling in asFP595 were undertaken in [27, 91] using other versions of the QM/MM method. The common TD-DFT and SA-CASSCF approaches were applied to calculate the points on the potential energy surfaces of the ground and excited states within a relatively small quantum subsystem. In [91], forces computed "on the fly" were used for trajectory calculations, and transitions between potential surfaces were allowed upon photoisomerization of the chromophore inside the protein. The main result of this modeling is a conclusion about the coupling of the trans-cis isomerization of the chromophore in the protein asFP595 with the protonation state of the chromophore. Similar technical approaches assuming trajectory calculations with forces estimated "on the fly" by quantum equations were used for an analysis of GFP photodynamics [73]. In a series of papers [92-96], the results of molecular dynamics simulations of proton transfers over hydrogen bond networks in the vicinity of the chromophore in GFP were presented using various presentations of the potential surfaces, including those by the quantum calculations.

In the very first applications of the QM/MM method for calculating the properties of fluorescent proteins [50, 51], an essentially simpler - but less reliable - methodology was applied. According to it, the structural parameters of the protein molecule were obtained with the semiempirical quantum chemistry approach AM1 and the excitation energies were estimated in the TD-DFT approximation. By using this methodology, the bands in the optical spectra of GFP [50] (as well as those of the blue fluorescent protein BFP [51], in which the GFP chromophore was modified) were estimated. In the actively cited paper [97], the positions of bands in optical spectra of GFP corresponding to the transitions S0 → S1 and S1 → S0 were computed in the QM/MM approach using the ab initio CASSCF method in the quantum subsystem and the force field CHARMM in the MM subsystem. The computed band positions are consistent with the experimental results, giving rise to discrepancies of about 20-30 nm. By considering a series of models with gradually increasing quantum subsystems, the authors studied the effect of the charged amino acid residue Arg located near the chromophore (Fig. 9) on the calculated spectrum.

The optical spectra of GFP and several mutated variants with different protonation states of chromophore groups were computed in [98]. To calculate the energy differences of the ground and excited states, the authors used a specific version of the configuration interaction method as in previous studies of the gas phase chromophores [56], but they accounted for the effect of the protein matrix within the QM/ MM approach. Good agreement between the calculated and experimental transition energies both for excitation and emission was reported.

The use of one of the so-called multi-level quantum chemistry approaches for treating extended systems - the fragment molecular orbital (FMO) method - to calculate the optical spectra of the red fluorescent proteins DsRed and mFruits was described in [99, 100]. The results were obtained within various versions of the configuration interaction approximation for estimates of the energy differences between the ground and excited states. The FMO method is potentially interesting due to the possibility of avoiding the use of the empirical force fields, avoiding the combined QM/MM approach in calculations of protein properties, and applying only quantum chemical approximations to the model system.

## CONCLUSIONS

Over the ten years since the publication of the first papers [12, 31, 33] devoted to the computer modeling properties of fluorescent proteins and their chromophores, a large amount of results have been obtained, most of which were discussed in this review. Apparently, the greatest interest is in answering the question as to what the experimenters can learn from the results of computer modeling that is particularly useful. Let us turn to one of the recent review papers written by experts in the studies of fluorescent proteins, Tonge and Meech [7]. They draw attention to several computational papers that they selected, which are mentioned below. An analysis of the electronic structure of the chromophore molecule, hydroxybenzylidene-imidazoline, in the ground and excited electronic states performed in the first calculations using the semiempirical methods of quantum chemistry [32, 33] allowed to relate the photophysical properties of GFP with the local properties of the bridging fragment of the molecule (Fig. 2). In particular, increasing the bond order of the methylene′s double bond upon electronic excitation should lead to the internal rotation barrier decreasing and facilitate internal conversion and benefit the trans-cis chromophore isomerization. The importance of calculating the sections of the potential energy surfaces, the minimum energy pathways along the angular coordinates near the bridging fragment, and the conical intersection points for the chromophore molecule in the isolated state and in solution upon the gradually increasing complexity of the quantum chemistry approaches [57, 60, 61, 69, 72, 91, 101] is underlined. Since such calculations with an explicit consideration of the role of the protein matrix on the photophysical properties of the chromophore are too complicated, several modeling results [21, 22] obtained with molecular mechanics methods are distinguished (in particular, those that formulated the role of sterical hindrance for the internal conversion of the chromophore). As is shown in QM/ MM calculations [97], the charged amino acid residue may considerably affect the photoexcitation dynamics. Molecular dynamics simulations (sometimes in conjunction with quantum chemistry calculations) [16, 28, 64, 92-96, 102, 103] allow one to visualize the proton transfer pathways along oriented hydrogen bond networks in proteins or transformations with the chromophore groups. The latter observations seem to be important for a prognosis of perspective point mutations, which may either enhance or diminish these pathways.

Therefore, the entire range of modern tools of computer molecular modeling, including molecular mechanics, molecular dynamics, quantum chemistry, and combined-quantum mechanics and molecular-mechanics (QM/MM) methods - all of which were used for modeling the structure and spectra of fluorescent proteins -described in this review are considered in [7] as useful support in experimental studies that are, in turn, oriented toward the practically important tasks of designing new and efficient biomarkers in living systems by the directed modification of natural objects [104].

We consider modeling with the QM/MM method the most prospective, but the most time-consuming tool for simulations of chemical and photophysical phenomena in proteins. Future success in this direction depends on how progress in the construction of supercomputers goes; on the development of efficient algorithms to solve the equation of quantum mechanics; and, even to a larger extent, on the existence of qualified specialists capable of understanding a wide range of subjects from biology to computational mathematics. These efforts will be granted if reliable predictions of perspective variants of protein macromolecules can be provided quickly to biotechnologists at least as efficiently as computer modeling turned out to be useful in drug design [105].

## Acknowledgements

When preparing this article, works supported by the Russian Foundation of Basic Research (project # 07-03-00059) and the Program of the Russian Academy of Sciences on molecular and cell biology and the Federal Science and Innovation Agency (project 02.522.11.2002) were used.
